# Supplementary material for: Chronic cold exposure enhances glucose oxidation in brown adipose tissue
Source: EMBO Rep. 2020 Oct 12;21(11):e50085. doi: 10.15252/embr.202050085 (PMC7645266; doi:10.15252/embr.202050085)
Supplement: Supplementary file 1 — Appendix [file EMBR-21-e50085-s001.pdf]

Chronic cold exposure enhances glucose oxidation in brown adipose tissue

Short title: Cold enhances glucose oxidation in BAT

Zhichao Wang[1,5], Tinglu Ning[1,5], Anying Song[1], Jared Rutter[3,4], Qiong A. Wang[1,2]*, Lei Jiang[1,2]*

[1]Department of Molecular & Cellular Endocrinology, Diabetes and Metabolism Research Institute, [2]Comprehensive Cancer Center, Beckman Research Institute, City of Hope Medical Center, Duarte, California 91010, USA. [3]Howard Hughes Medical Institute, [4]Department of Biochemistry, University of Utah School of Medicine, Salt Lake City, Utah 84112, USA. [5]Co-first authors

Appendix Supplementary content:

Appendix Figure S1, Chronic cold exposure induces oxidative metabolism in BAT. Page 2

Appendix Figure S2, Chronic cold exposure induces oxidative metabolism in sWAT. Page 3

Appendix Figure S3, Chronic cold exposure induces oxidative metabolism in female mice. Page 4

Appendix Table S1, β3-AR agonist induces oxidative metabolism in brown adipocytes. Page 5

Appendix Table S2, Data used for metabolic flux analysis (MFA) Page 7

Appendix Table S3, The primers for real-time PCR. Page 9

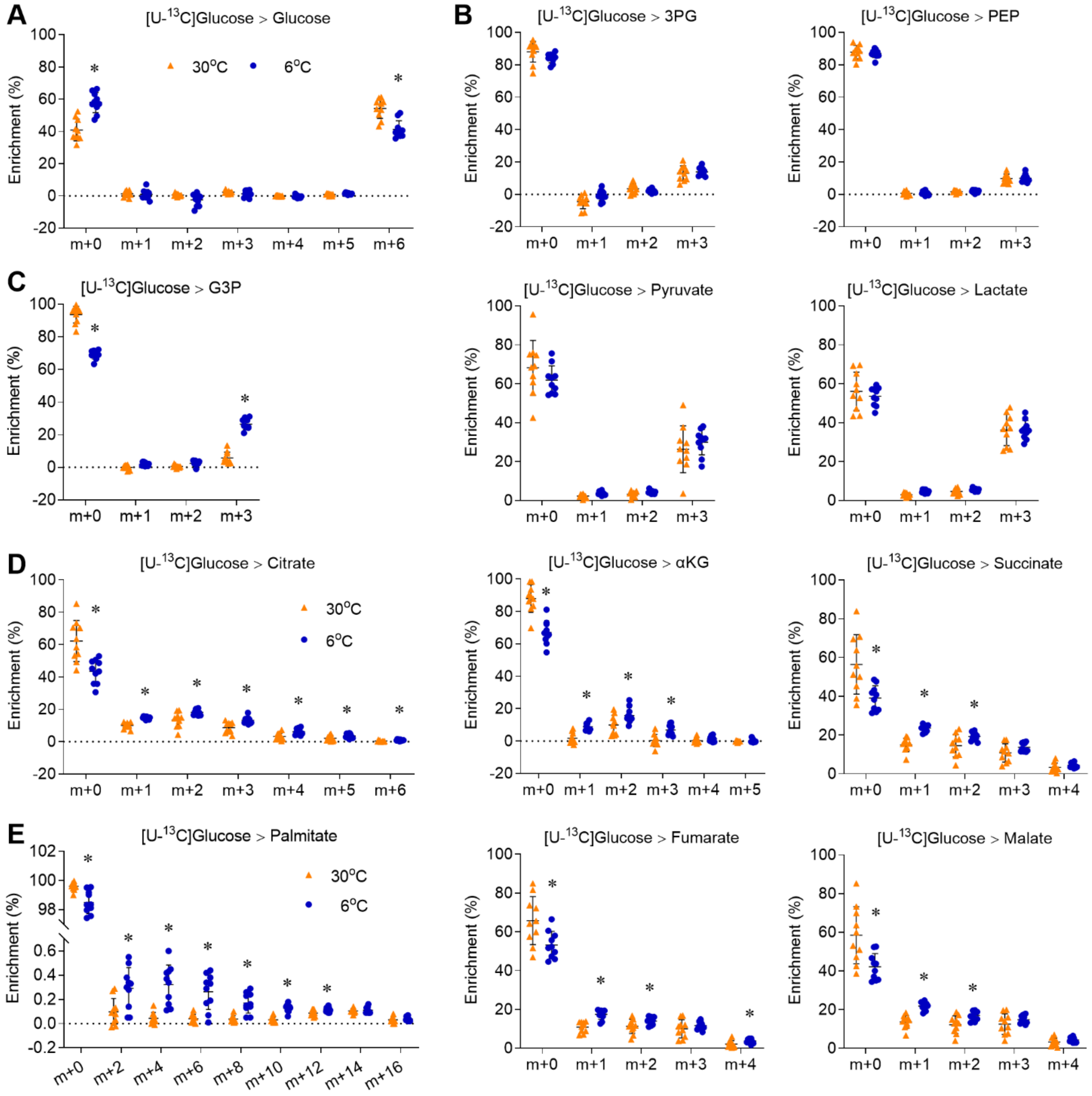

Appendix Figure S1, Chronic cold exposure induces oxidative metabolism in BAT. Mice, housed at 30°C or 6°C for 10 days, were administered with [U-^{13}C]glucose (2 g/kg, IP). 15 minutes after injection, BAT was harvested for metabolic enrichment assay. The full enrichments of the metabolites in BAT are shown here, and the major metabolite isotopologues are presented in **Figure 1 and EV1.** n=10, Data are represented as the mean ± s.d. Statistical analysis was performed using one-way ANOVA followed by Tukey multiple comparisons, *P < 0.05.

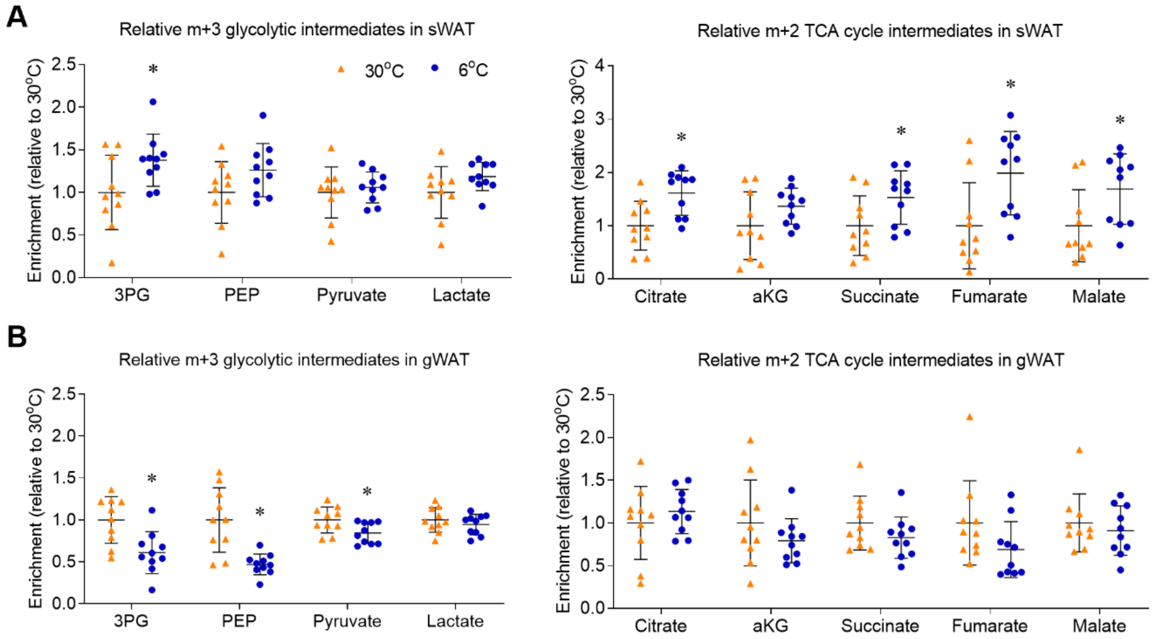

Appendix Figure S2, Chronic cold exposure induces oxidative metabolism in sWAT. This figure is related to figure 2, and the data are calculated from the same data set as figure 2. Mice, housed at 30°C or 6°C for 10 days, were administered with [U-^{13}C]glucose (2 g/kg, IP). 15 minutes after injection, sWAT and gWAT were harvested for metabolic enrichment assay. (**A**) To directly compare the change of enrichment between different metabolites, the ^{13}C enrichments in sWAT of male mice are normalized to the average enrichment of each metabolite in 30°C group and shown as relative enrichment. (**B**) The ^{13}C enrichments in gWAT of male mice are normalized to the average enrichment of each metabolite in 30°C group and shown as relative enrichment. n=10, Data are represented as the mean ± s.d. Statistical analysis was performed using two-tailed Student's t-test, *P < 0.05.

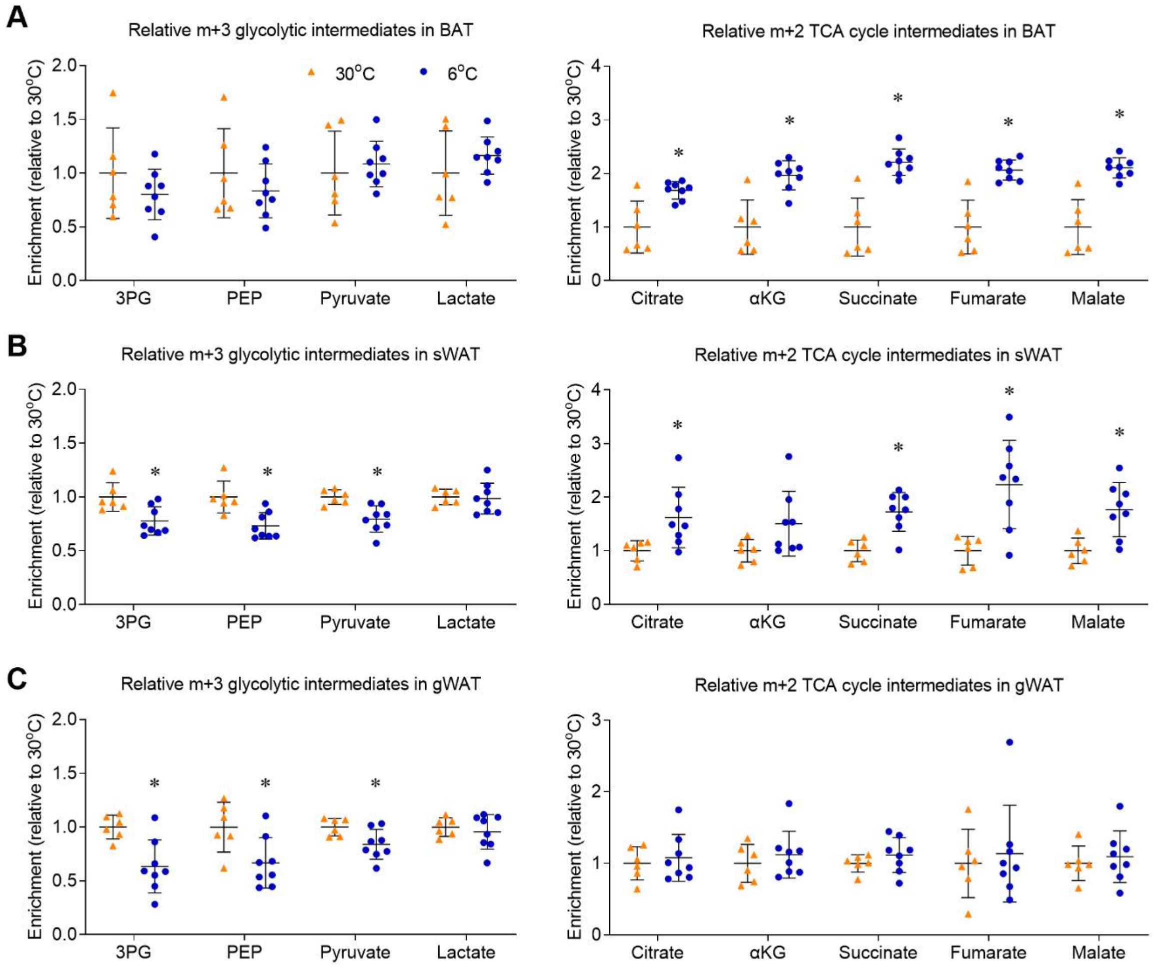

Appendix Figure S3, Chronic cold exposure induces oxidative metabolism in BAT and sWAT of female mice. Female mice, housed at 30°C or 6°C for 10 days, were administered with [U-^{13}C]glucose (2 g/kg, IP). 15 minutes after injection, BAT, sWAT and gWAT were harvested for metabolic enrichment assay. To directly compare the change of enrichment between different metabolites, the ^{13}C enrichments in BAT(**A**), sWAT (B), gWAT (C) of female mice are normalized to the average enrichment of each metabolite in 30°C group and shown as relative enrichment. n=6~8, Data are represented as the mean ± s.d. Statistical analysis was performed using two-tailed Student's t-test, *P < 0.05.

Appendix Table S1, β3-AR agonist induces oxidative metabolism in brown adipocytes.

	Net Flux	Day6		CL		
	Reaction	Value	95% C.I.	Value	95% C.I.	Ratio
Glc uptake	Glc.x -> Glc.c	0.4700	[0.4608,0.4792]	1.9700	[1.9314,2.0086]	4.19
Glycolysis	Glc.c -> Pyr.c + Pyr.c	0.4700	[0.4608,0.4792]	1.9700	[1.9314,2.0086]	4.19
Pyr dilution	Pyr.d -> Pyr.c	0.4426	[0.2375,0.5961]	1.1123	[0.6535,1.3177]	2.51
LDH	Pyr.c -> Lac.c	0.8400	[0.8235,0.8565]	3.1200	[3.0589,3.1812]	3.71
Lac secretion	Lac.c -> Lac.x	0.8400	[0.8235,0.8565]	3.1200	[3.0589,3.1812]	3.71
Pal dilution	Pal.d -> Pal.c	0.1724	[0.1502,0.1983]	0.3367	[0.2977,0.3804]	1.95
Pal transport	Pal.c -> Pal.m	0.1744	[0.1522,0.2003]	0.3387	[0.2997,0.3824]	1.94
Beta oxidation	Pal.m -> 8*AcCoA.m	0.1744	[0.1522,0.2003]	0.3387	[0.2997,0.3824]	1.94
MPC	Pyr.c -> Pyr.m	0.7081	[0.6081,0.8246]	2.0317	[1.8152,2.3549]	2.87
PDH	Pyr.m -> AcCoA.m + CO2.out	0.2834	[0.2339,0.3321]	1.1592	[1.0191,1.3202]	4.09
CS	AcCoA.m + OAA.m -> Cit.m	1.6790	[1.5001,1.8778]	3.8691	[3.4844,4.2900]	2.30
IDH	Cit.m -> αKG.m + CO2.out	1.6630	[1.4841,1.8618]	3.8531	[3.4684,4.2740]	2.32
OGDH	αKG.m -> Suc.m + CO2.out	1.4037	[1.1422,1.6950]	3.0800	[2.6617,3.8335]	2.19
SDH	Suc.m -> Fum.m	1.4037	[1.1422,1.6950]	3.0800	[2.6617,3.8335]	2.19
FH	Fum.m <-> Mal.m	1.4037	[1.1422,1.6950]	3.0800	[2.6617,3.8335]	2.19
MDH.m	Mal.m <-> OAA.m	1.2543	[1.0863,1.4322]	2.9966	[2.6689,3.3609]	2.39
PC	Pyr.m + CO2.x -> OAA.m	0.4247	[0.3347,0.5354]	0.8725	[0.7370,1.0957]	2.05
Gln dilution	Gln.d -> Gln	1.7063	[1.4767,1.9790]	3.4129	[3.0613,3.8684]	2.00
GLS	Gln <-> Glu	1.7063	[1.4767,1.9790]	3.4129	[3.0613,3.8684]	2.00
Glu secretion	Glu -> Glu.out	1.9656	[1.7547,2.2447]	4.1860	[3.7502,4.6140]	2.13
GDH	αKG.m <-> Glu	0.2592	[0.0466,0.4218]	0.7731	[0.2464,0.9638]	2.98
CTP	Cit.m <-> Cit.c	0.0160	[0.0160,0.0160]	0.0160	[0.0160,0.0160]	1.00
ACLY	Cit.c -> AcCoA.c + OAA.c	0.0160	[0.0160,0.0160]	0.0160	[0.0160,0.0160]	1.00
MDH.c	OAA.c <-> Mal.c	0.0160	[0.0160,0.0160]	0.0160	[0.0160,0.0160]	1.00
FASN	8*AcCoA.c -> Pal.c	0.0020	[0.0020,0.0020]	0.0020	[0.0020,0.0020]	1.00
ME	Mal.c -> Pyr.c + CO2.out	0.1654	[0.0000,0.4634]	0.0994	[0.0000,0.7983]	0.60
Mal transport	Mal.m <-> Mal.c	0.1494	[-0.016,0.4474]	0.0834	[-0.016,0.7823]	0.56

Appendix Table S1, β3-AR agonist induces oxidative metabolism in brown adipocytes. (Continue)

	Exchanging	Day6		CL	
	Reaction	Value	95% C.I.	Value	95% C.I.
FH	Fum.m <-> Mal.m	10.770	[3.8454,Inf]	14.885	[5.1453,1225600]
GLS	Gln <-> Glu	0.4559	[0.0483,1.1116]	0.4044	[0.0000,0.9507]
GDH	αKG.m <-> Glu	211440	[12.9068,Inf]	100000	[65.286,100000]
CTP	Cit.m <-> Cit.c	0.0001	[0.0000,Inf]	0.0000	[0.0000,Inf]
MDH.m	Mal.m <-> OAA.m	56.167	[3.4935,Inf]	7.2191	[3.2464,17.4905]
MDH.c	OAA.c <-> Mal.c	0.0112	[0.0000,Inf]	0.0022	[0.0000,Inf]
Mal transport	Mal.m <-> Mal.c	0.0000	[0.0000,NaN]	0.1146	[0.0000,NaN]

	Mixing	Day6		CL	
	Reaction	Value	95% C.I.	Value	95% C.I.
v1	0*Cit.m -> Cit.mix	0.8436	[-4.4409E-16,1]	0.0789	[-5.5511E-16,1]
v2	0*Cit.c -> Cit.mix	0.1564	[0.0000,1.0000]	0.9211	[0.0000,1.0000]
v3	Cit.mix -> sink	1.0000	[1.0000,1.0000]	1.0000	[1.0000,1.0000]
v4	0*Mal.c -> Mal.mix	1.0000	[-5.5511E-16,1]	0.9999	[-4.4409E-16,1]
v5	0*Mal.m -> Mal.mix	0.0000	[0.0000,1.0000]	0.0001	[0.0000,1.0000]
v6	Mal.mix -> sink	1.0000	[1.0000,1.0000]	1.0000	[1.0000,1.0000]
v7	0*Pyr.c -> Pyr.mix	1.0000	[-2.2204E-16,1]	1.0000	[-2.2204E-16,1]
v8	0*Pyr.m -> Pyr.mix	0.0000	[0.0000,1.0000]	0.0000	[0.0000,1.0000]
v9	Pyr.mix -> sink	1.0000	[1.0000,1.0000]	1.0000	[1.0000,1.0000]

Flux was determined as mentioned at method. Glucose uptake rates and lactate secretion rates were calculated by detection of medium glucose and lactate concentration before and after treatment. Negative values mean the direction of the bidirectional reaction is reverse with the indicated direction. The sum of squared residuals was 48.7 for DMSO and 41 for CL, both were acceptable (20.6~53.2). Both degrees of freedom were 35. The unit of flux ratio is nmol/h/μg protein.

Abbreviations: c, cytosolic; m, mitochondrial; Inf, infinity; C.I., Confidence interval.

Metabolites: AcCoA, Acetyl-CoA; αKG, α-ketoglutarate; Cit, citrate; Fum, fumarate; Glc, glucose; Gln, glutamine; Glu, glutamate; Lac, lactate; Mal, malate; OAA, oxaloacetic acid; Pal, palmitate; Pyr, pyruvate; Suc, succinate.

Enzymes and transporters: ACLY, ATP citrate lyase; CS, citrate synthase; CTP, citrate transporter protein; FASN, fatty acid synthase; FH, fumarase; GDH, glutamate dehydrogenase; GLS, glutaminase; IDH, isocitrate dehydrogenase; LDH, lactate dehydrogenase; MDH, malate dehydrogenase; ME, malic enzyme; MPC, mitochondrial pyruvate carrier; OGDH, α-ketoglutarate dehydrogenase; PC, pyruvate carboxylase; PDH, pyruvate dehydrogenase; SDH, succinate dehydrogenase.

Appendix Table S2, Data used for metabolic flux analysis (MFA).

	DMSO	CL
Glc uptake (nmol/h/ug protein)	0.47	1.97
Lac secretion (nmol/h/ug protein)	0.84	3.12
Pyr enrichment		
M+0	0.35	0.22
M+1	0.01	0.01
M+2	0.02	0.00
M+3	0.62	0.76
Lac enrichment		
M+0	0.39	0.24
M+1	0.01	0.01
M+2	0.04	-0.03
M+3	0.56	0.77
Cit enrichment		
M+0	0.65	0.47
M+1	0.04	0.06
M+2	0.14	0.20
M+3	0.12	0.13
M+4	0.02	0.06
M+5	0.02	0.05
M+6	0.02	0.03
αKG enrichment		
M+0	0.84	0.73
M+1	0.01	0.02
M+2	0.10	0.16
M+3	0.03	0.04
M+4	0.01	0.04
M+5	0.00	0.02
Gln enrichment		
M+0	0.97	0.97
M+1	0.00	0.00
M+2	0.02	0.02
M+3	0.01	0.01
M+4	0.00	0.00
M+5	0.00	0.00
Fum enrichment		
M+0	0.73	0.66
M+1	0.04	0.06
M+2	0.09	0.13
M+3	0.13	0.11
M+4	0.02	0.04

Appendix Table S2, Data used for metabolic flux analysis (MFA). (Continue)

Suc enrichment		
M+0	0.81	0.71
M+1	0.05	0.06
M+2	0.12	0.15
M+3	0.01	0.03
M+4	0.01	0.05
Mal enrichment		
M+0	0.72	0.64
M+1	0.04	0.06
M+2	0.09	0.13
M+3	0.14	0.14
M+4	0.01	0.04
Glu432 enrichment		
M+0	0.84	0.71
M+1	0.02	0.03
M+2	0.10	0.16
M+3	0.03	0.04
M+4	0.01	0.04
M+5	0.00	0.02
Glu330 enrichment		
M+0	0.84	0.72
M+1	0.02	0.04
M+2	0.12	0.17
M+3	0.00	0.02
M+4	0.01	0.04
Pal enrichment		
M+ 0	1.00	0.97
M+ 1	0.00	0.02
M+ 2	0.00	0.00
M+ 3	0.00	0.00
M+ 4	0.00	0.00
M+ 5	0.00	0.00
M+ 6	0.00	0.00
M+ 7	0.00	0.00
M+ 8	0.00	0.00
M+ 9	0.00	0.00
M+10	0.00	0.00
M+11	0.00	0.00
M+12	0.00	0.00
M+13	0.00	0.00
M+14	0.00	0.00
M+15	0.00	0.00
M+16	0.00	0.00

Appendix Table S3, The primers for real-time PCR.

	Forward primer 5’ – 3’	Reverse primer 5’ – 3’
Ucp1	AGGCTTCCAGTACCATTAGGT	CTGAGTGAGGCAAAGCTGATTT
Elovl3	TTCTCACGCGGGTTAAAAATGG	GAGCAACAGATAGACGACCAC
Cidea	TGACATTCATGGGATTGCAGAC	GGCCAGTTGTGATGACTAAGAC
Prdm16	CCACCAGCGAGGACTTCAC	GGAGGACTCTCGTAGCTCGAA
Pgc-1α	TATGGAGTGACATAGAGTGTGCT	CCACTTCAATCCACCCAGAAAG
Pparγ2	TCGCTGATGCACTGCCTATG	GAGAGGTCCACAGAGCTGATT
Dio2	AATTATGCCTCGGAGAAGACCG	GGCAGTTGCCTAGTGAAAGGT
Mpc1	ATGAGTACGCACTTCTGGGG	CGCCCACTGATAATCTCTGGA
Mpc2	TACCACCGACTCATGGATAAAGT	CACACACCAATCCCCATTTCA
Rplpo	AGATTCGGGATATGCTGTTGGC	TCGGGTCCTAGACCAGTGTTC
