# Supplementary material for: Chronic cold exposure enhances glucose oxidation in brown adipose tissue
Source: EMBO Rep. 2020 Oct 12;21(11):e50085. doi: 10.15252/embr.202050085 (PMC7645266; doi:10.15252/embr.202050085)
Supplement: Supplementary file 2 — Expanded View Figures PDF [file EMBR-21-e50085-s002.pdf]

Expanded View Figures

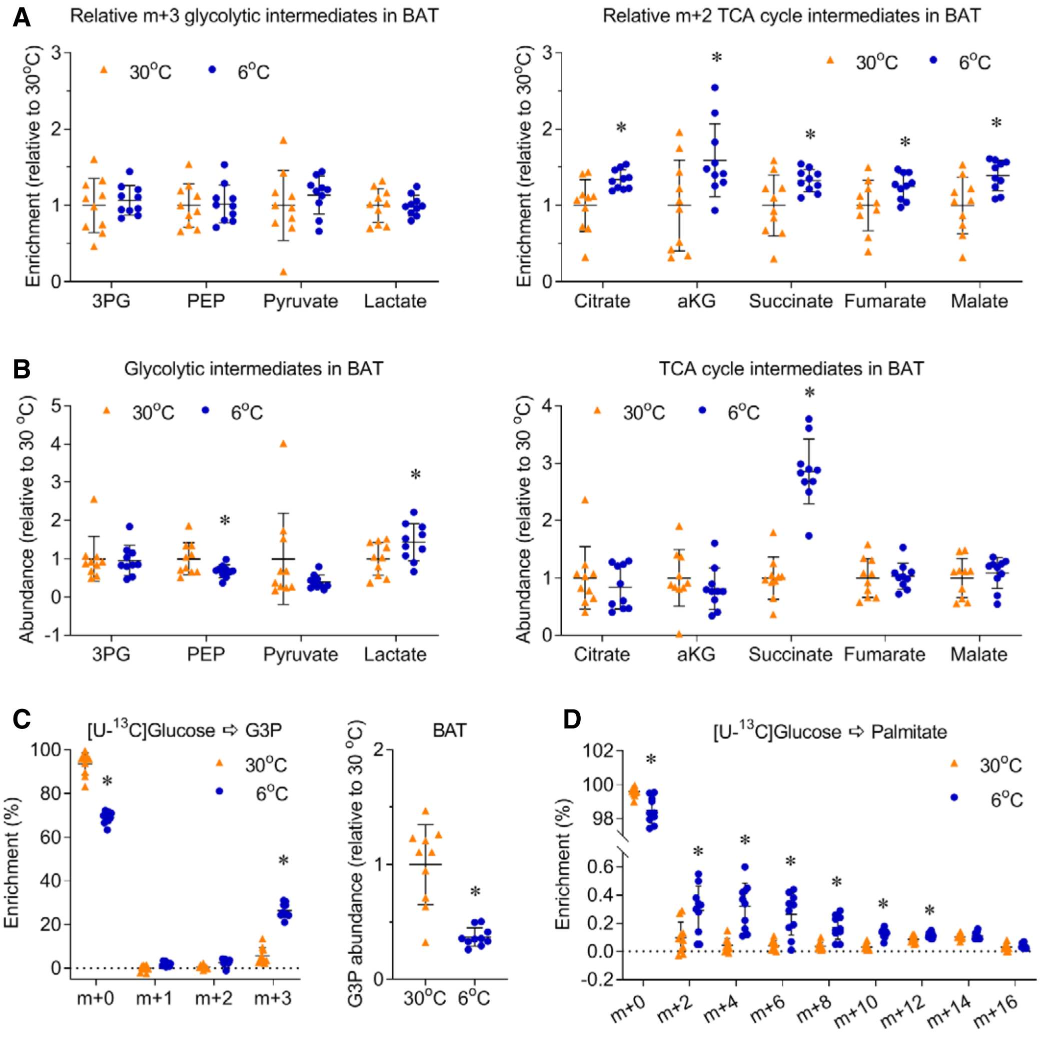

Figure EV1. Chronic cold exposure induces glucose metabolism in BAT.

Mice, housed at 30°C or 6°C for 10 days, were administered with [U-^{13}C]glucose (2 g/kg, IP). 15 minutes after injection, BAT was harvested for metabolic enrichment assay.

A To directly compare the change of enrichment between different metabolites, the relative metabolic ^{13}C enrichments in BAT of male mice are normalized to the average enrichment of each metabolite in 30°C group.
B Relative abundance of glycolytic and TCA cycle intermediates in BAT.
C The enrichment and relative abundance of glyceraldehyde 3-phosphate (G3P).
D The enrichment of palmitate.

Data information: $n = 10$, data are represented as the mean ± SD. Statistical analysis was performed using two-tailed Student's *t*-test, *$P < 0.05$.
Source data are available online for this figure.

 © 2020 The Authors

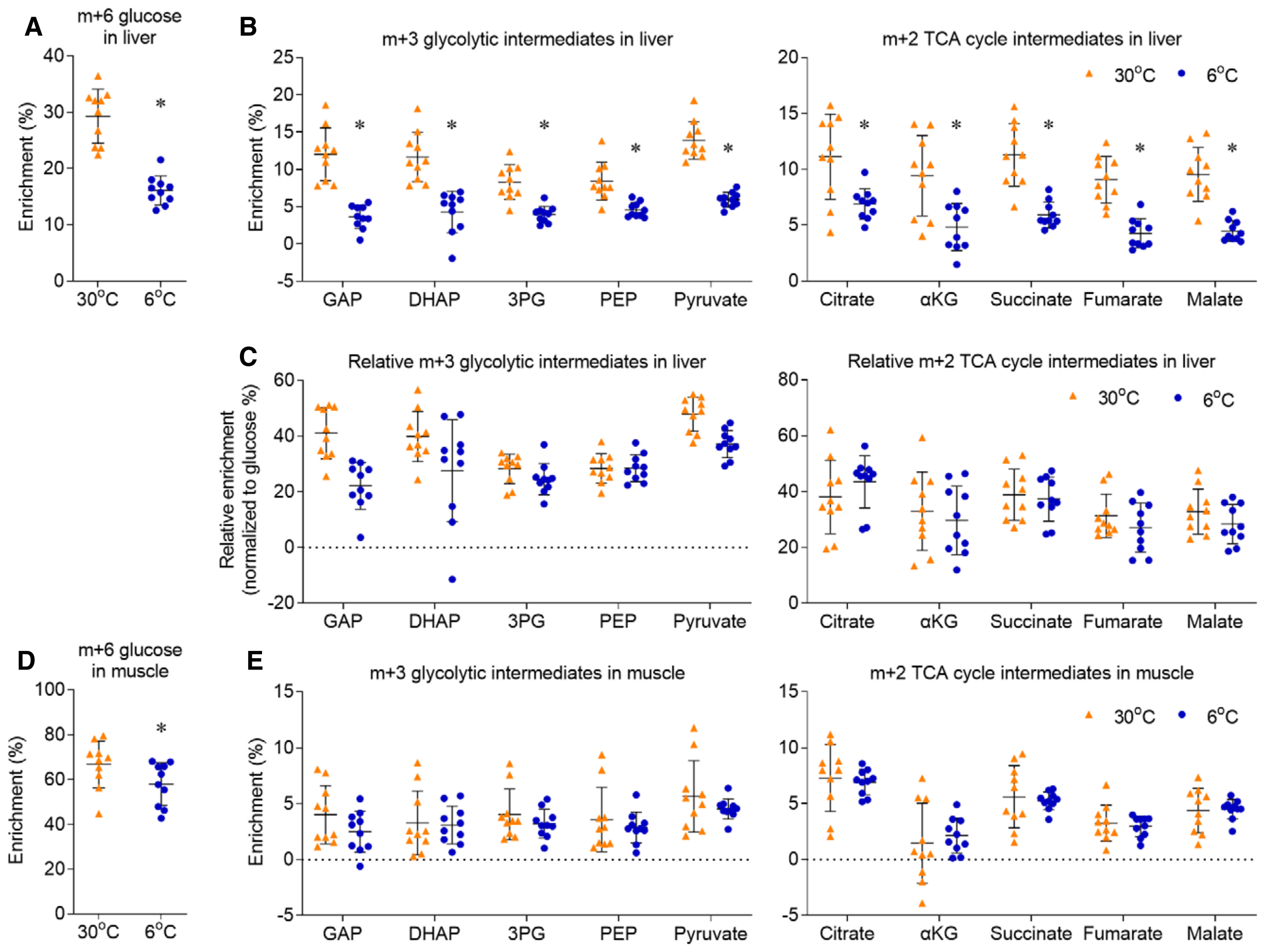

Figure EV2. Chronic cold exposure does not induce oxidative metabolism in liver or muscle.

Mice, housed at 30°C or 6°C for 10 days, were administered with [U-^{13}C]glucose (2 g/kg, IP). 15 minutes after injection, liver and muscle were harvested for metabolic enrichment assay.

- A m+6 glucose enrichment in liver.
- B Metabolic ^{13}C enrichments in liver are shown as m+3 glycolysis intermediates, m+2 TCA cycle intermediates. GAP, glyceraldehyde 3-phosphate; DHAP, dihydroxyacetone phosphate.
- C After normalizing to the glucose enrichment in the liver of each mouse, the relative metabolic ^{13}C enrichments were shown as m+3 glycolysis intermediates and m+2 TCA cycle intermediates.
- D m+6 glucose enrichment in muscle.
- E Metabolic ^{13}C enrichments in muscle are shown as m+3 glycolysis intermediates, m+2 TCA cycle intermediates.

Data information: n = 10, data are represented as the mean ± SD. Statistical analysis was performed using two-tailed Student's t-test, *$P < 0.05$.

Source data are available online for this figure.

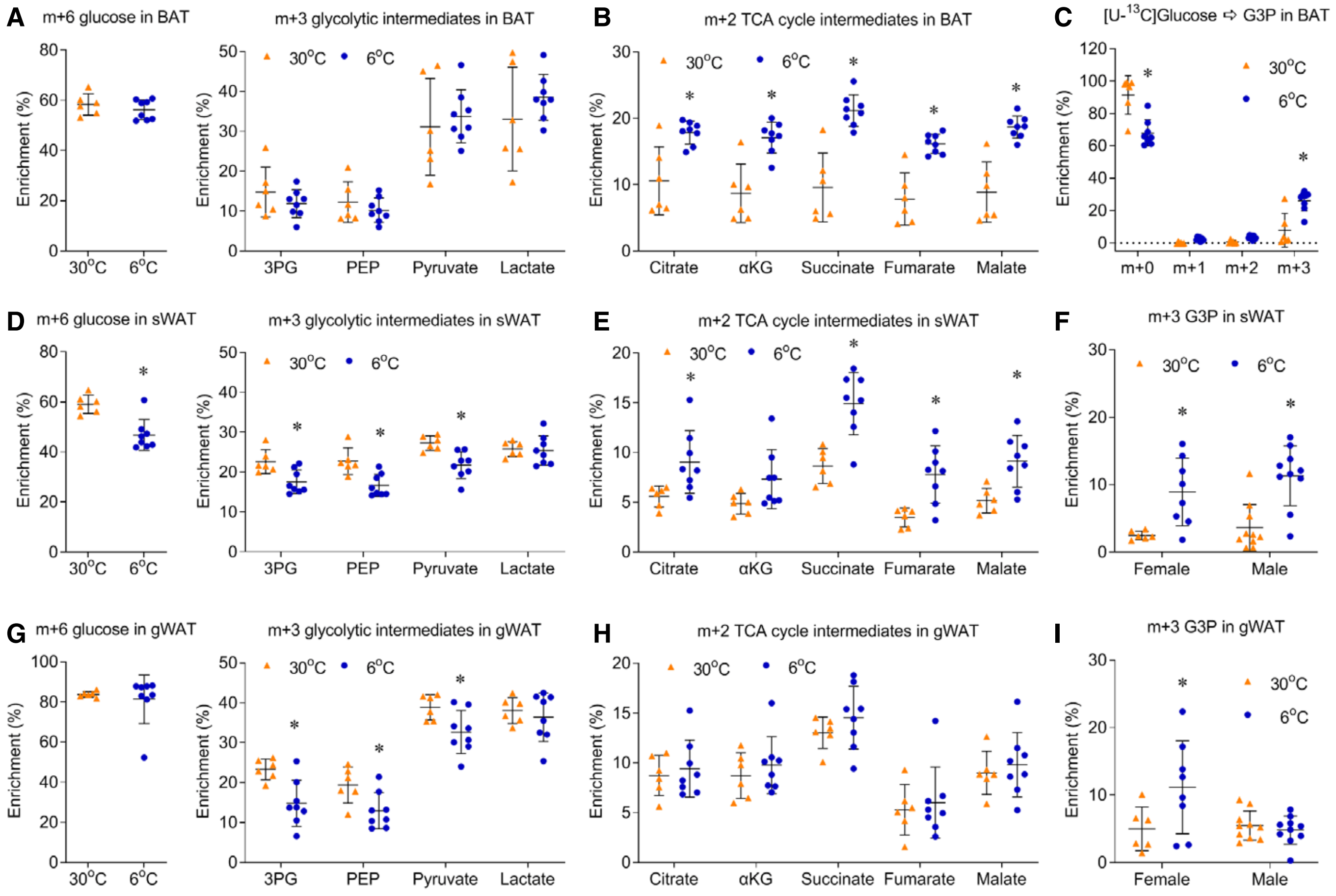

Figure EV3. Chronic cold exposure induces oxidative metabolism in BAT and sWAT of female mice.

Mice, housed at 30 or 6°C for 10 days, were administered with [U-^{13}C]glucose (2 g/kg, IP). 15 minutes after injection, BAT, sWAT, and gWAT were harvested for metabolic enrichment assay.

A–C Metabolic ^{13}C enrichments in BAT of female mice are shown as m+6 glucose and m+3 glycolytic intermediates (A), m+2 TCA cycle intermediates (B), and the enrichment of G3P (C).

D, E Metabolic ^{13}C enrichments in sWAT of female mice are shown as m+6 glucose and m+3 glycolysis intermediates (D), m+2 TCA cycle intermediates (E).

F The m+3 enrichment of G3P in sWAT of both female and male mice.

G, H Metabolic ^{13}C enrichments in gWAT of female mice are shown as m+6 glucose and m+3 glycolysis intermediates (G), m+2 TCA cycle intermediates (H).

I The m+3 enrichment of G3P in gWAT of both female and male mice.

Data information: n = 6–8 female mice, and n = 10 male mice, data are represented as the mean ± SD. Statistical analysis was performed using two-tailed Student's t-test, *$P < 0.05$.

Source data are available online for this figure.

 © 2020 The Authors

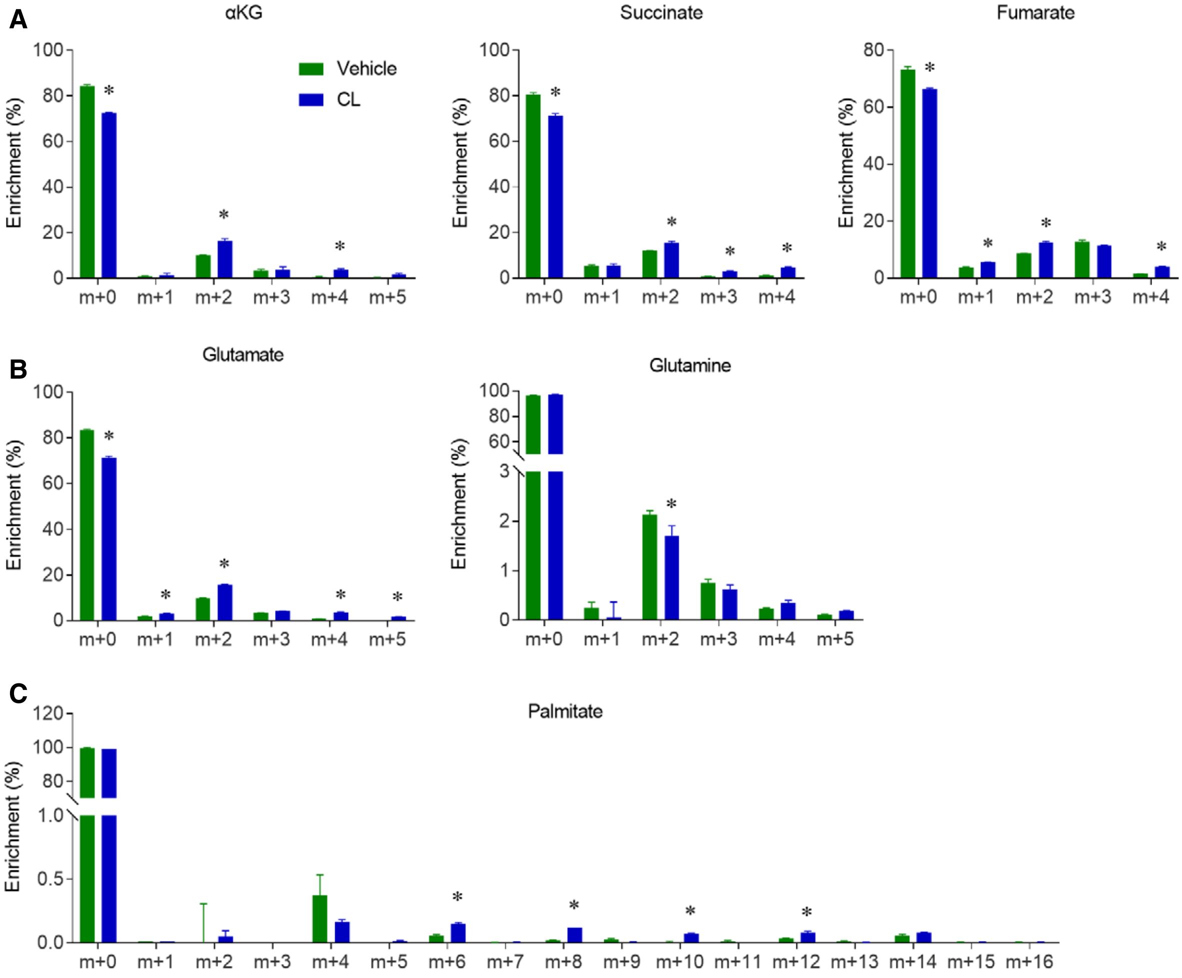

Figure EV4. β3-AR agonist activates glucose oxidation in differentiated primary brown adipocytes.

A–C In the sample [U-^{13}C]glucose experiment as shown in Fig 5, the enrichments of other metabolites were used for MFA modeling. *n* = 3 biological repeats, data are represented as the mean ± SD. Statistical analysis was performed using two-way ANOVA followed by Tukey's multiple comparisons test, *$P < 0.05$.

Source data are available online for this figure.

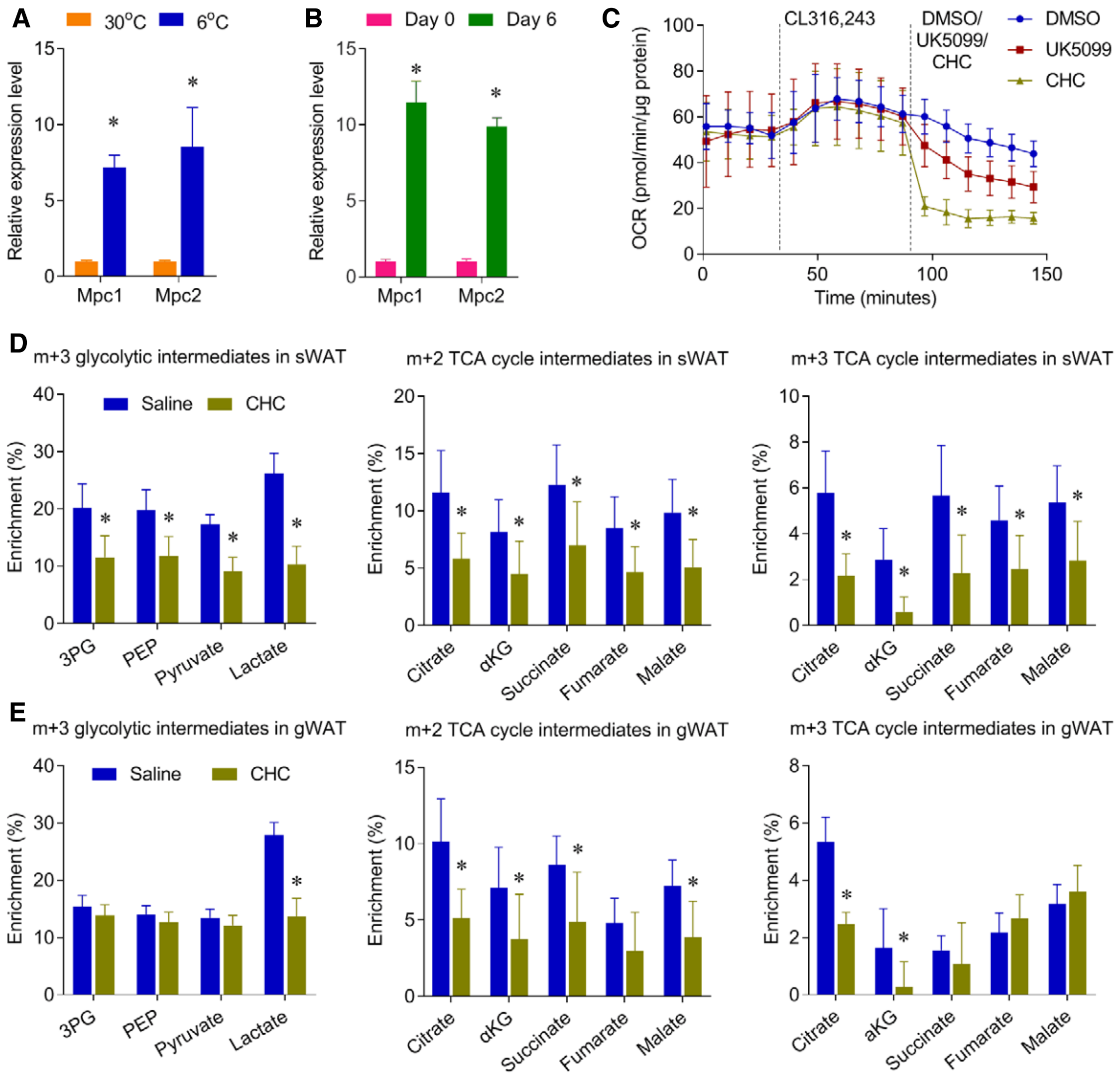

Figure EV5. CHC represses glucose metabolism in multiple adipose tissues.

A Relative mRNA levels of Mpc1/2 expression were measured by qPCR in BAT of the mice, housed at 30 or 6°C for 10 days. *n* = 5–6 biological replicates.

B Relative mRNA levels of Mpc1/2 expression were measured by qPCR in the pre-differentiated day 0 and fully differentiated brown adipocytes day 6. *n* = 4 biological replicates.

C Oxygen consumption rate (OCR) of mouse brown adipocytes treated with MPC inhibitor CHC (2 mM) or UK5099 (2 μM), *n* = 6–7 biological repeats. CL, CL316,243.

D, E Mice were housed at 6°C for 10 days, and mice were IP injected with PBS or CHC (500 mg/kg). 30 minutes after CHC treatment, mice were administered with [U-^{13}C]glucose (2 g/kg, IP). Metabolic ^{13}C enrichments in sWAT (D) and gWAT (E) of male mice are shown as m+2 and m+3 TCA cycle intermediates. *n* = 7 biological replicates.

Data information:, data are represented as the mean ± SD, except that (C) is represented as the mean ± SEM. Statistical analysis was performed using two-tailed Student's *t*-test, *$P < 0.05$.

Source data are available online for this figure.

 © 2020 The Authors
